# Effect of Surgical Removal of Endometriomas on Cyclic and Non-cyclic Pelvic Pain

**DOI:** 10.22074/ijfs.2015.4252

**Published:** 2015-07-27

**Authors:** Murat Api, Aysen Telce Boza, Semra Kayatas, Mustafa Eroglu

**Affiliations:** Zeynep Kamil Women and Children Diseases Training and Research Hospital, Department of Obstetrics and Gynecology, Istanbul, Turkey

**Keywords:** Endometrioma, Pelvic Pain, Endometriosis, Ablation

## Abstract

**Background:**

Endometriosis is a complex disease with a spectrum of pain symptoms from mild dysmenorrhea to debilitating pelvic pain. There is no concrete evidence in the literature whether endometriotic cyst per se, causes pain spectrum related to the disease. The aim of the present study was to evaluate the effect of surgical removal of endome- triomas on pain symptoms.

**Materials and Methods:**

In this prospective, observational, before-after study, which was conducted between March 2012 and January 2013 in Training and Research Hospital,Adana, Turkey, a total of 23 patients including 16 sexually active and 7 vir- gin symptomatic women were questioned for non-cyclic pelvic pain (NCPP), intensity of the NCPP, presence of cyclic dysmenorrhea, and dyspareunia before and after the endometrioma operation. Participants who were sonographically diagnosed and later pathologically confirmed as having endometrioma without sign and symptoms of deep infiltrative endometriosis (DIE) were also questioned for pain symptoms before and after the laparoscopic removal of cyst wall. Patients with intraabdominal adhesions, history of pelvic inflammatory disease, and pathological diagnosis other than endometrioma were excluded. No ancillary procedures were applied for pain management, but if pain was present, pelvic peritoneal endometriotic lesions were ablated beside the removal of ovar- ian endometriotic cysts.

**Results:**

Out of 23 cases with endometrioma, 91 and 78% reported to have NCPP and dysmenorrhea, respectively, before the operation, while 60 and 48%, respec- tively, after the operation (McNemar’s test, P=0.016 for both figures). Among the sexually active cases, 31% (5/16) had dyspareunia before the operation and only 1 case reported the pain relief after the operation (McNemar’s test, P=1). Intensity of NCPP were reported to be none (8.7%), moderate (21.7%), severe (56.5%) and un- bearable (13%) before the operation and decreased to none (43.5%), mild (43.5%), moderate (4.3%) and severe (8.7%) after the operation (Wilcoxon signed-rank test, P<0.001).

**Conclusion:**

In symptomatic cases with ovarian endometrioma, without sign and symptoms of DIE, laparoscopic removal of the cysts with/without ablation of the peritoneal endometriotic lesions yields relief of NCPP and cyclic dysmenorrhea, but not dyspareunia.

## Introduction

Endometriosis is the most common gynecological pathology causing cyclic or non-cyclic pelvic pain (NCPP) accounting for 12-32% of women of reproductive age and for 45-70% in adolescents (1). Endometriosis is defined as the presence of endometrial glands and stroma outside of the endometrial cavity. Pelvic pain has long been recognized as a critical concomitant of the endometriosis syndrome. Up to 75% of symptomatic endometriosis causes cyclic pelvic pain with menstruation (2), though it is often associated with several different pain symptoms including noncyclical, nonmenstrual pelvic pain (3, 4). Indeed, in Sampson’s treatise (5), 12 of the 17 symptomatic cases he reported presented for surgery due to intolerable pain. As long as 90 years, the relationship between the extent of adhesion and severity of pain has not been well recognized. This lack of correlation continues to confound modern era gynecologists in large part (6) because mediation of painful stimuli are inadequately understood.

Microscopic studies have documented nerve fibres in endometriotic peritoneal lesions (7-9), deep infiltrating endometriosis (10, 11) and ovarian endometriomas (12). Berkley et al. (13) described the growth of efferent sympathetic and afferent sensory nerves into the ectopic implants of endometriosis in women and in a rat model of disease. The studies about neurogenesis in endometriosis caught the attention of gynecologists, physiologists and neuroscientists to evaluate the causes and develop new methods with transdisciplinary effort to ameliorate pain associated with endometriosis. On the other hand, surgery has long been an important part of the management of endometriosis. In 2011, Stratton and Berkley (14), described current approaches to surgical treatment of endometriosis based on "oncological principle" to remove all visible lesions and restore normal anatomy. Endometriomas are not amenable to medical treatment and need to be removed surgically even if symptoms improve with medical treatment (15), while the preferred therapeutic approach for women with symptomatic endometriomas was surgery to relieve the patient’s pain (16). Regardless of the stage of endometriosis, randomized controlled trials comparing the effect of surgery to conservative management have shown that surgery and excision of endometriosis results in symptomatic improvement (17, 18). The purpose of the present study was to discuss the benefits of surgical treatment for different types of pain associated with endometriomas.

## Materials and Methods

In a prospective, observational, before-after study, was conducted between March 2012 and January 2013 in Adana Numune Training and Research Hospital, Adana, Turkey. Twenty three cases including 16 sexually active and 7 virgin women (mean age: 31.9, range: 20-43) who were sonographically diagnosed and later pathologically confirmed as having unilateral endometrioma (3-8 cm in diameter) without sign and symptoms of deep infiltrative endometriosis (DIE), as dyschezia, hematuria, rectal bleeding, constipation, diarrhea and bloating, formed our study group. The patients whose rectal/ rectovaginal examination, imaging studies [ultrasound and magnetic resonance imaging (MRI)], and intraoperative findings suggested DIE as well as the patients who had intra-abdominal adhesions, history of pelvic inflammatory disease, and pathological diagnosis other than endometrioma were all excluded. Women completed a preoperative questionnaire that collected demographic characteristics and data on presenting problem as full menstrual history, medical and surgical history and characteristics of pain symptoms. Pain was assessed using a verbal scale which has good correlation with visual analogue scale and higher compliance in clinical settings (19). The verbal scale offered descriptors such as "no pain, moderate pain, severe pain, and unbearable pain". Women were questioned for pain symptoms preoperatively and 3-6 months after the laparoscopic removal of endometrioma.

All patients were operated using stripping method by the same operator. No ancillary procedures as presacral neurectomy, uterosacral interruptions of sensory nerves and uterine suspension were applied for pain management, but if pain was present, pelvic peritoneal endometriotic lesions were ablated beside the removal of ovarian endometriotic cysts. The diagnosis of endometrioma was confirmed by histological examination of specimens removed at surgery.

### Statistical analysis

Pain relief was analyzed by the McNemar’s test for pre- and post-operative symptoms. Analyses of pain scores were performed using the Wilcoxon signed-rank test for paired non parametric data. Analyses was undertaken Statistics Package for the Social Sciences (SPSS, SPSS Inc. Chicago, IL, USA) version 15. A P value of <0.05 was accepted as statistically significant. Qualitative data are expressed in percentage (%) and quantitative data are expressed as the means ± standard deviation (SD).

The study protocol was elaborated according to the revised Declaration of Helsinki and was approved by the Local Research and Ethics Committee of Adana Numune Training and Research Hospital in Adana, Turkey. All subjects were provided a written informed consent.

## Results

Average age of women at the time of surgery was 31.9 (range 20-43). Out of 23 subjects, 43.5% (10/23) were nulliparous and 56.5% were parous. Average endometrioma cyst diameter were 43.7 ± 21.7 mm. Twenty two women had no previous abdominal procedure; only one woman had a laparoscopic endometrioma ablation before (Table 1).

**Table 1:** The demographic characteristics of the patients


Mean age (range), Y	31.9 (20-43)
Parity, n (%)	
Nulliparity	10 (43.5%)
Multiparity	13 (56.5%)
Sexual behaviour, n (%)	
Sexually active	16 (69.5%)
Virgin	7 (30.4%)
Previous abdominal procedure, (%)	1 (4.34%)
Average endometrioma cyst diameter (mm), mean ± SD	43.7 ± 21.7
Postoperative medical therapy, n (%)	0 (0%)


mm; Millimeter.

**Fig.1:**
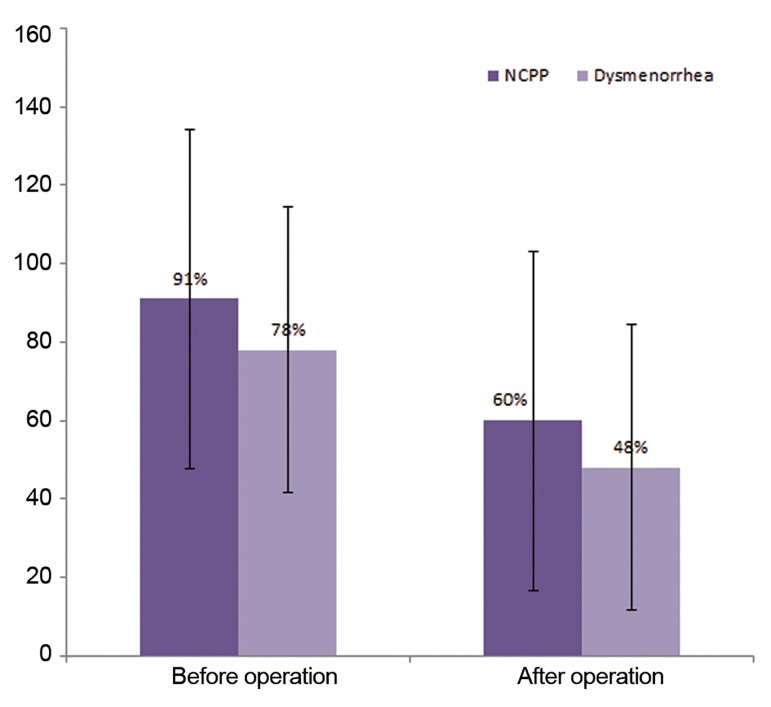
Percentages and %95 confidence intervals (CI) for noncyclic pelvic pain (NCPP) and dysmenorrhea before and after the operation.

Out of 23 cases with unilateral endometrioma, 91% (21/23) reported to have NCPP before the operation, but this ratio decreased to 60% after the operation (McNemar’s test P=0.016). The frequency of dysmenorrhea was also felt by 30% after the operation (78 to 48%, McNemar’s test P=0.016, Fig.1).

**Fig.2:**
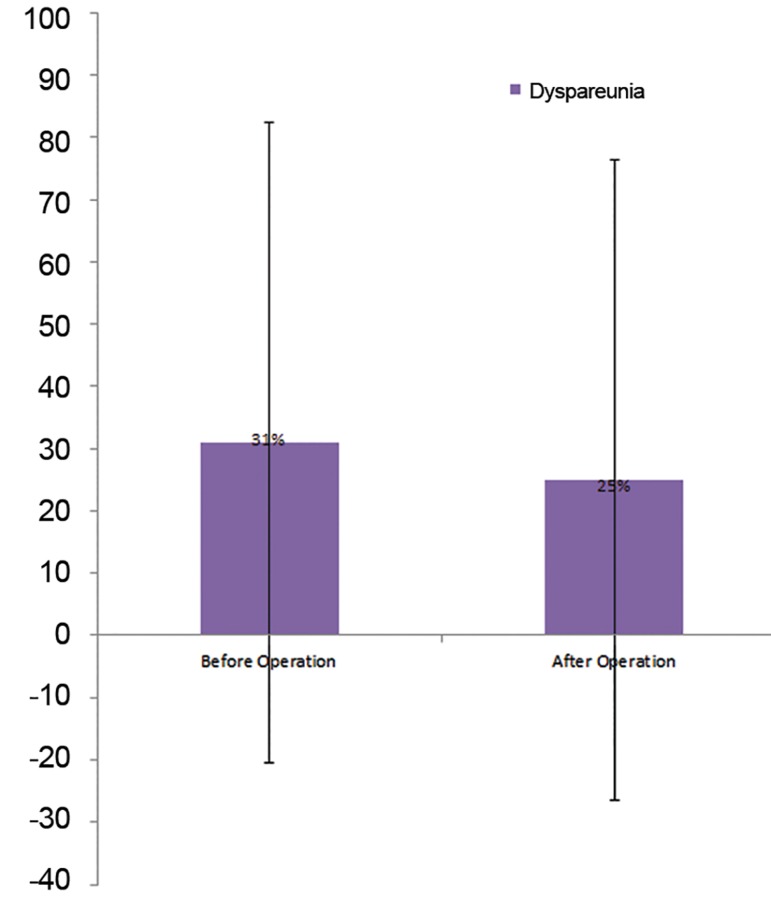
Percentages and %95 confidence intervals (CI) for dyspareunia before and after the operation.

Among the sexually active cases, 31% (5/16) had dyspareunia before the operation and only 1 case reported pain relief after the operation (Mc- Nemar’s test P=1, Fig.2).

**Fig.3:**
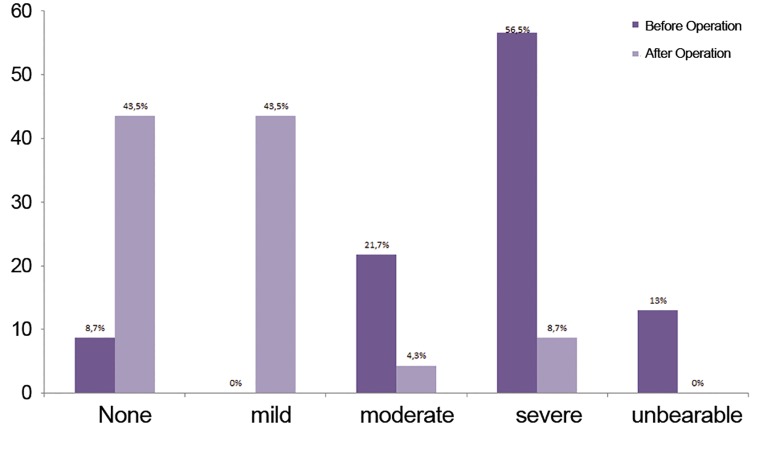
Pain grades for non-cyclic pelvic pain (NCPP) before and after the operation.

Intensity of NCPP were reported to be none (8.7%), moderate (21.7%), severe (56.5%) and unbearable (13%) before the operation and decreased to none (43.5%), mild (43.5%), moderate (4.3%) and severe (8.7%), after the operation (Wilcoxon signed-rank test P<0.001, Fig.3).

Nine of 23 patients had mild lesions on peritoneal surfaces and were ablated by bipolar cautery. None of the patients were scheduled for long term pain management.

## Discussion

Women with endometriosis either may have diverse and nonspecific symptoms or may be asymptomatic. The prevalance of endometriosis in asymptomatic women in general population are not known, but pain is the most common symptom associated with endometriosis, diagnosed by visulization of pelvic organs via laparoscopy. Approximately three quarters of symptomatic patients experience nonmenstrual pelvic pain and/or dysmenorrhea (20). In the present study, all participants had different types of pain as follows: 91% had NCPP, 78% had dysmenorrhea, and 21.7% had dyspareunia.

According to the current guideline by European Society of Human Reproduction and Embryology (ESHRE 2013) (21), asymptomatic endometriosis that is incidentally diagnosed should not be operated. Both surgical and medical treatments show improvements in pain scores of symptomatic cases. However, there is no published trials directly comparing one againist the other; therefore, we must rely on other evidence to weigh up the pros and cons of each approach. Unlike medical treatments, surgery can diagnose and remove all macroscopic disease at the same procedure in the majority of cases. In the case of symptomatic endometrioma, suggested and preffered therapeutic approach is surgery. Medical therapy is unlikely to result in complete regression of endometriomas larger than 1 cm and precludes a definitive histologic diagnosis (22, 23).

There have been very few studies in the current literature evaluating the effect of removal of endometrioma on pain symptoms. The efficacy of surgical management of endometriosis was demonstrated by a randomized trial, comparing the outcome of women after therapeutic laparoscopy with the outcome of women who underwent diagnostic laparoscopy alone. Laparoscopic excision of implants led to symptomatic improvement in 80% of patients at six months compared to 32% of controls undergoing diagnostic laparoscopy (3). Ideally if the surgery is performed for diagnosis, consent has to be obtained for surgical resection/ ablation of endometriosis at the same time (20).

According to a review by Jadoul et al. (24) in which they analyzed the arguments in favour of and against of surgical treatments of endometriosis and showed that more than 50% of the patients reported pain relief. Also the operation technique used for endometrioma removal affects the pain relief. Several techniques have been described to treat endometriomas. In most of these techniques, the procedure consists of opening and draining the cyst followed by either excision (stripping technique), fulguration, or vaporisation of the cystic wall (ablative technique) (25-28). Drainage is alone not recommended because of the high recurrence rate (29). Hart’s Cochrane systematic review found that excisional surgery provides better improvement in pain scores and decreases chance of recurrence compared with ablation (30). In our study the stripping technique was used, while the incidence and severity of NCPP and dysmenorrhea were significantly improved after the operation, as similiar to these studies. Only dyspareunia symptom was remained following the surgery. Ovary is one of the most frequent location for endometriosis, leading to the extensive pelvic and intestinal disease. Caution must be paid not to underdiagnose or undertreat these women (31). Although we tried to exclude the DIE preoperatively by asking symptoms and performing rectovaginal/rectal and imaging examinations and our operative findings also excluded DIE, we still thought that the presence of endometriotic invisible lesions caused dyspareunia. Milingos et al. (32) found that symptoms of deep dyspareunia was correlated with the presence of dense pelvic adhesions and related to advanced endometriosis. The stripping and ablating of endometriotic lesions seemed to be not enough to improve dyspareunia. On the other hand, the nature of endometriotic pain was reported to be memorized in the brain that might not be resolved by excision of endometrioma. The possible explanation of remaining dyspareunia after the endometrioma excision could be the painful intercourse memorized by the certain brain area (14).

There were some limitations in our study. Due to small number of participants, we were unable to categorized the subjects according to the size of endometriomas, although no larger study was conducted on this subject yet.

## Conclusion

One hundred and fifty years after endometriosis was first described, we are still debating both its etiology and management. Although main questions remain unanswered, solid evidence shows that laparoscopic surgery appears to be the most logical approach to treatment. As a result of our study, we want to emphasize that in cases of symptomatic endometriosis, without sign and symptoms of DIE, laparoscopic removal of the cysts with/without ablation of the peritoneal endometriotic lesions may relive NCPP and cyclic dysmenorrhea, but not dyspareunia.

## References

[1] Laufer MR, Goitein L, Bush M, Cramer DW, Emans SJ (1997). Prevalence of endometriosis in adolescent girls with chronic pelvic pain not responding to conventional therapy. J Pediatr Adolesc Gynecol.

[2] Howard F, Howard FM, Perry CP, Carter JE, El-Minawi AM (2000). Endometriosis and endosalpingiosis. Pelvic pain: diagnosis and management.

[3] Abbott J, Hawe J, Hunter D, Holmes M, Finn P, Garry R (2004). Laparoscopic excision of endometriosis: a randomized, placebo-controlled trial. Fertil Steril.

[4] Fedele L, Bianchi S, Bocciolone L, Di Nola G, Parazzini F (1992). Pain symptoms associated with endometriosis. Obstet Gynecol.

[5] Sampson JA (1921). Perforating hemorrhagic (chocolate) cysts of the ovary. Arch Surg.

[6] Hoeger KM, Guzick DS (1999). An update on the classification of endometriosis. Clin Obstet Gynecol.

[7] Tokushige N, Markham R, Russell P, Fraser IS (2006). Nerve fibres in peritoneal endometriosis. Hum Reprod.

[8] Anaf V, Simon P, El Nakadi I, Fayt I, Simonart T, Buxant F (2002). Hyperalgesia, nerve infiltration and nerve growth factor expression in deep adenomyotic nodules, peritoneal and ovarian endometriosis. Hum Reprod.

[9] Mechsner S, Kaiser A, Kopf A, Gericke C, Ebert A, Bartley J (2009). A pilot study to evaluate the clinical relevance of endometriosis-associated nerve fibers in peritoneal endometriotic lesions. Fertil Steril.

[10] Anaf V, Simon P, El Nakadi I, Fayt I, Buxant F, Simonart T (2000). Relationship between endometriotic foci and nerves in rectovaginal endometriotic nodules. Hum Reprod.

[11] Wang G, Tokushige N, Markham R, Fraser IS (2009). Rich innervation of deep infiltrating endometriosis. Hum Reprod.

[12] Tokushige N, Russell P, Black K, Barrera H, Dubinovsky S, Markham R (2010). Nerve fibers in ovarian endometriomas. Fertil Steril.

[13] Berkley KJ, Rapkin AJ, Papka RE (2005). The pains of endometriosis. Science.

[14] Stratton P, Berkley KJ (2011). Chronic pelvic pain and endometriosis: translational evidence of the relationship and implications. Hum Reprod Update.

[15] Adamson GD, Subak LL, Pasta DJ, Hurd SJ, von Franque O, Rodriguez BD (1992). Comparison of CO2 laser laparoscopy with laparotomy for treatment of endometrioma. Fertil Steril.

[16] Abbott JA, Hawe J, Clayton RD, Garry R (2003). The effects and effectiveness of laparoscopic excision of endometriosis: a prospective study with 2-5 year follow-up. Hum Reprod.

[17] Sutton CJ, Pooley AS, Ewen SP, Haines P (1997). Follow-up report on a randomized controlled trial of laser laparoscopy in the treatment of pelvic pain associated with minimal to moderate endometriosis. Fertil Steril.

[18] Jarrell J, Mohindra R, Ross S, Taenzer P, Brant R (2005). Laparoscopy and reported pain among patients with endometriosis. J Obstet Gynaecol Can.

[19] Briggs M, Closs JS (1999). A descriptive study of the use of visual analogue scales and verbal rating scales for the assessment of postoperative pain in orthopedic patients. J Pain Symptom Manage.

[20] Sinaii N, Plumb K, Cotton L, Lambert A, Kennedy S, Zondervan K (2008). Differences in characteristics among 1,000 women with endometriosis based on extent of disease. Fertil Steril.

[21] Dunselman G, Vermeulen N, Nelen W (2013). The 2013 ESHRE guideline on the management of women with endometriosis. Hum Reprod.

[22] Alborzi S, Zarei A, Alborzi S, Alborzi M (2006). Management of ovarian endometrioma. Clin Obstet Gynecol.

[23] Chapron C, Vercellini P, Barakat H, Vieira M, Dubuisson JB (2002). Management of ovarian endometriomas. Hum Reprod Update.

[24] Jadoul P, Kitajima M, Donnez O, Squifflet J, Donnez J (2012). Surgical treatment of ovarian endometriomas: state of the art?.. Fertil Steril.

[25] Canis M, Rabischong B, Houlle C, Botchorishvili R, Jardon K, Safi A (2002). Laparoscopic management of adnexal masses: a gold standard?.. Curr Opin Obstet Gynecol.

[26] Reich H, McGlynn F (1986). Treatment of ovarian endometrioma using laparoscopic surgical techniques. J Reprod Med.

[27] Sutton CJ, Jones KD (2002). Laser laparoscopy for endometriosis and endometriotic cysts. Surg Endosc.

[28] Jones KD, Sutton CJ (2000). Laparoscopic management of ovarian endometriomata: a critical review of current practice. Curr Opin Obstet Gynecol.

[29] Donnez J, Nisolle M, Gillerot Setal, Anaf V, Clerckx-Braun F, Casanas-Roux F (1994). Ovarian endometrial cysts: the role of gonadotropin-releasing hormone agonist and/or drainage. Fertil Steril.

[30] Hart RJ, Hickey M, Maouris P, Buckett W (2008). Excisional surgery versus ablative surgery for ovarian endometriomata. Cochrane Database Syst Rev.

[31] Redwine DB (1999). Ovarian endometriosis: a marker for more extensive pelvic and intestinal disease. Fertil Steril.

[32] Milingos S, Protopapas A, Kallipolitis G, Drakakis P, Loutradis D, Liapi A (2006). Endometriosis in patients with chronic pelvic pain: is staging predictive of the efficacy of laparoscopic surgery inpain relief?.. Gynecol Obstet Invest.

